# The Story of the Insane from Year to Year

**Published:** 1901-11-16

**Authors:** 


### THE STORY OF THE INSANE FROM YEAR TO YEAR.

(Thirteenth Series.)

### WEXFORD DISTRICT ASYLUM AT ENNISCORTHY.

The limits of accommodation are stated to be 448 beds. In Table I. there is a misprint of "1898" for "1899"; but it would seem that the asylum contained 421 patients at the beginning of the year 1899, and that it contained 442 at the end of that year. There were 70 first admissions, 27 readmissions, 43 recoveries, 4 discharged relieved, and 28 deaths. The daily average number resident was 435, and the total number of persons under treatment during the year was 517. The percentage of recoveries on the admissions was 44·3, and that of the deaths on the average number resident was 6·4. Compared with English County Asylums the recoveries are high and the deaths low. Of the year's deaths 9 were caused by cerebral and spinal diseases, 6 by consumption, 2 by pneumonia, and 2 by dysentery. Various moral causes account for the insanity in six of the patients admitted during the year, while of the physical causes hereditary influence stands first with a total of 47 out of 91 admissions, and drink is credited with being the cause in 16 cases. Here, then, we have in this single district the discreditable fact disclosed that in at least 70 per cent. of the year's admissions the insanity was preventible. Dr. Drapes says that he "need not dwell further on the subject except to emphasise the fact that both causes, but especially drink, are to a large extent preventible and ought as far as possible to be eliminated if the public, in whose hands this lies, is at all in earnest in its desire to confine the disease within its narrowest limits." We gladly give a wider circulation to these remarks than they would have if left in an asylum report, but we sadly fear that the public are not in earnest in this matter, and they take no action partly because they are not aware of the enormous development of the evil and partly because they doubt whether any Government would take the matter up and forbid the marriage of those having hereditary insanity. The annual cost per head was £27 7s. 5d.

### CUMBERLAND AND WESTMORLAND ASYLUM.

On January 1st, 1899, this asylum contained 651 patients, and on December 31st the number had fallen to 645. There were 131 first admissions, 35 readmissions, 91 recoveries, 31 discharged relieved, and 44 deaths. The average number resident during the year was 642, and the total number of persons under care was 805. The recoveries, calculated on the year's admissions stand at 54·8 per cent., and the deaths calculated on the average number resident stand at 6·8 per cent., the former being as much above the usual recovery rate as the latter is below the usual death-rate. Of the year's deaths only 5 were caused by cerebral or spinal diseases, but there were 15 from consumption, 2 from pneumonia, and 1 from pleurisy. Ulcerative colitis claimed 1 victim. Turning to Table X. which shows the assigned causes of insanity in the admissions of the year, we find that mental anxiety was the cause in 14 instances, domestic trouble in 6, religion in 4, intemperance in drink in 25, and hereditary influence in 51. Adding the two last causes together it will be seen that they accounted for nearly 46 per cent. of the admissions—a state of things very terrible to look at, and unfortunately by no means confined to Cumberland and Westmorland. The deaths from lung mischief were certainly high, and Dr. Farquharson says: "The contagious nature of phthisis is now clearly recognised, and large asylums afford great opportunities for the spread of the disease, many of the insane are so uncleanly in their habits that it is impossible to carry out thoroughly the precautions necessary for preventing the dissemination of the tubercle bacillus unless there be some means whereby all those already suffering from the disease can be isolated from their fellow-patients." These are very sensible and opportune remarks, but it does not seem that any action is being taken to provide a sanatorium for this purpose. We notice, however, that the committee have handed over to the County Councils from the maintenance account a sum of £4,000. If this sum has been saved from the profits on out-county and private patients it is a perfectly correct transaction, but it would have been advisable to spend £1,000 of it in putting up a sanatorium. If, however, the money were saved from in-county patients it is very questionable whether it could be used either for this purpose or handed over to the council. The committee say that "the efforts made by the medical superintendent and the staff of officials to effect the recovery of the patients are most satisfactory." The weekly charge per head is 8s. 9d. There are about 50 private patients, and these pay from 14s. to 17s. 6d.

### THE CARMARTHEN ASYLUM.

On January 1st, 1899, this asylum contained 618 patients, and on December 31st the number had risen to 650. There were 94 first admissions, 15 readmissions, 21 recoveries, 2 discharged relieved, 8 discharged not improved, and 46 deaths. The average number resident during the year was 630, and the total number of persons under treatment was 724. The recoveries stand at 22·8 per cent. of the admissions, and the deaths at 7·36 per cent. on the average number resident. Of the year's deaths 15 were caused by cerebral and spinal diseases, 11 by consumption, 4 by pneumonia, and 2 by colitis. Of the year's admissions 18 were due to domestic trouble, mental anxiety, religion, love, etc., 15 to intemperance in drink, and 30 to hereditary influence. Dr. Goodall says that after "making due allowance for the various causes of apparent increase in the volume of officially-known insanity, the opinion is now freely expressed by those most competent to judge that insanity is really increasing." This certainly agrees with the views which have been expressed more than once in this journal; but we had thought that official authority was against us. There seems no loophole for escape from the unwelcome conclusion. Referring to the death-rate Dr. Goodall advises the treatment of phthisical cases in separate blocks, and he adds that "no doubt these measures will be provided for in asylums of the future." Why not at present? Wooden buildings would be better than brick or stone, and could be run up quickly and cheaply. A case of that rare disease known as acute ascending paralysis was under treatment. The patient had been in his usual health; was suddenly seized with paralysis in the legs: the paralysis soon involved the arms and the swallowing apparatus, and the patient died four days after the appearance of the first symptoms. "The most curious point about the disease is that the nervous system shows nothing morbid on minute examinations. It appears to be due to some acute toxic influence." The committee "testify to the efficient management of the institution under Dr. Goodall." The weekly charge per head for Carmarthen patients is 7s. 7d. There are about 50 out-county patients, and the charge for these is 13s. to 14s. The private patients pay from 10s. to 42s.

